# Ligation of the Pancreatic Stump With Quantified Force During Distal Pancreatectomy for Postoperative Pancreatic Fistula: Protocol for a Single-Center Nonrandomized Controlled Clinical Study

**DOI:** 10.2196/74018

**Published:** 2025-07-08

**Authors:** Lufeng Chang, Jiongxin Xiong, Ming Yang, Yuxin Yang, Tao Peng, Tao Yin, Heshui Wu, Shanmiao Gou

**Affiliations:** 1 Department of Pancreatic Surgery, Union Hospital Tongji Medical College Huazhong University of Science and Technology Wuhan China; 2 Sino-German Laboratory of Personalized Medicine for Pancreatic Cancer, Union Hospital Tongji Medical College Huazhong University of Science and Technology Wuhan China

**Keywords:** pancreatic surgery, distal pancreatectomy, pancreatic stump ligation, quantified ligation force, postoperative pancreatic fistula

## Abstract

**Background:**

The incidence of postoperative pancreatic fistula following distal pancreatectomy is as high as 30%-50%. Postoperative pancreatic fistula can be a major cause of perioperative morbidity, resulting in prolonged hospital stays and increased health care costs. The management of the pancreatic stump is one of the key factors influencing the occurrence of postoperative pancreatic fistula after distal pancreatectomy, but the optimal management approach remains debatable. The main methods for pancreatic stump closure include manual suturing and stapler closure. However, both methods are associated with a high risk of postoperative pancreatic fistula, which may be related to the balance between providing sufficient pancreatic duct burst pressure and ensuring blood supply to the stump. Ligation of the pancreatic stump has been attempted to reduce the risk of postoperative pancreatic fistula following distal pancreatectomy, but its efficacy remains limited by the challenge of achieving the optimal ligation force.

**Objective:**

This study aims to investigate whether ligation of the pancreatic stump with a quantified force can decrease the risk of postoperative pancreatic fistula following distal pancreatectomy.

**Methods:**

In this nonrandomized controlled clinical study at a tertiary center in China, the major eligibility criterion is the presence of lesions planned for distal pancreatectomy. Sixty patients will be allocated to the experimental or control group according to their choice. Recruitment for either group will be discontinued upon reaching the predefined sample size of 30 participants. In the experimental group, the pancreas will be ligated 5 mm from the pancreatic stump with a quantified force to provide a pancreatic duct burst pressure of approximately 40-70 mm Hg. The ligation force will be provided by a 3.2-mm-diameter silicone ring. During pancreatic stump ligation, this silicone ring will be stretched to 15 mm, generating an applied force of 1.3 N. The pancreas will be severed using energy-based devices before or after the ligation. In the control group, the pancreatic stump will be managed by manual suturing or stapling closure according to the surgeon’s clinical judgment and preference. Postoperative regular follow-up examinations will be conducted. The primary outcomes include postoperative pancreatic fistula and postoperative hospital stay, and the secondary outcomes include intra-abdominal infection, incision infection, and postoperative treatment costs. The primary and secondary outcomes of patients in this cohort will be statistically compared using appropriate tests.

**Results:**

This study started in February 2025, and the recruitment period is from February to September 2025.

**Conclusions:**

This protocol proposes a novel approach for pancreatic stump management aimed at preventing postoperative pancreatic fistula following distal pancreatectomy. The research team established the optimal ligation force for the pancreatic stump to ensure adequate burst pressure for the pancreatic duct while preventing acute stump necrosis, thereby theoretically reducing the risk of postoperative pancreatic fistula.

**Trial Registration:**

Chinese Clinical Trial Register ChiCTR2500097781; https://www.chictr.org.cn/showproj.html?proj=247008

**International Registered Report Identifier (IRRID):**

DERR1-10.2196/74018

## Introduction

Distal pancreatectomy is the primary surgical procedure for treating inflammatory lesions as well as benign and malignant tumors in the pancreatic body and tail. Postoperative pancreatic fistula (POPF) is a common and severe complication of distal pancreatectomy, with an incidence rate of approximately 30%-50% [1,2]. POPF can lead to delayed gastric emptying, intestinal obstruction, hemorrhage, wound infection, intra-abdominal abscess, and sepsis. These complications can further increase hospital stay and medical expenses and may potentially result in death [3,4].

To reduce POPF, surgeons have explored numerous strategies, with the aim of achieving better outcomes through improved patient management and advanced techniques [5-10]. The closure technique used for the stump is a crucial factor influencing the occurrence of POPF, and 2 methods are primarily used at present: manual suturing and stapling closure [5,6]. Stapling devices are often used due to their simplicity and convenience, but they may not effectively close the pancreatic stump when the pancreas is hard or thick. In such cases, manual suturing is often the sole option. Additionally, surgeons have investigated techniques such as wrapping the stump with mesh [7], saline-coupled radiofrequency ablation [10], pancreaticojejunostomy [9], preoperative pancreatic duct stenting [11,12], and the application of fibrin patches to the stump [8]. Unfortunately, none of these techniques have significantly reduced the risk of pancreatic fistula. In their expert consensus on the management of the pancreatic stump during distal pancreatectomy in 2022, the International Study Group on Pancreatic Surgery stated that both manual suturing and stapling closure have their respective advantages, and both methods are associated with an equal risk of pancreatic fistula. The use of energy devices or additional biomaterials to seal the pancreas or the combined use of these methods also does not reduce the incidence of pancreatic fistula [13].

Closure of the main pancreatic duct (MPD) is a key step to prevent pancreatic fistula. However, stapling closure may not provide a sufficiently high burst pressure for the pancreatic duct, even when the pancreatic stump is adequately closed. Under physiological conditions, the pancreatic duct pressure ranges from 3 to 31 mm Hg [14,15], but the burst pressures provided by approximately half of the stapling devices for pancreatic duct closure are below 31 mm Hg [16,17], leading to pancreatic fistula in some patients. Manual suturing can provide a burst pressure of up to 400 mm Hg for the pancreatic duct through sutures, but the high pressure on the tissue can cause tissue necrosis and new pancreatic duct proliferation, resulting in pancreatic fistula [17]. Additionally, identification of MPD can be difficult after pancreatic transection in some patients, and empirical suturing increases the risk of pancreatic fistula.

Ligating the pancreatic stump is another technique for the closure of the pancreatic stump. Ligation of the pancreatic tissue proximal to the stump can provide sufficient burst pressure for the pancreatic duct. However, ligation may also lead to acute tissue necrosis and pancreatic duct regeneration, similar to suturing [18]. To address this issue, the project applicant quantified the ligation force to achieve a burst pressure of approximately 40-70 mm Hg for the pancreatic duct. This burst pressure is much higher than the maximum pressure of the pancreatic duct under physiological conditions but lower than the average arterial pressure under physiological conditions. Therefore, theoretically, the use of this burst pressure can avoid the direct leakage of the pancreatic juice from the pancreatic duct while reducing the risk of pancreatic stump acute necrosis by providing partial blood supply to the pancreatic stump, preventing POPF caused by the necrosis-proliferation process of the pancreatic stump.

Thus, we hypothesized that the rate of POPF in patients who undergo pancreatic stump ligation with a quantified force would be lesser than that using other closure techniques. To validate this hypothesis, we launched this clinical trial. According to the Evidence Map of Pancreatic Surgery, this is the first trial of its kind [19].

## Methods

### Study Design

The methods have been designed and are reported according to the SPIRIT (Standard Protocol Items: Recommendations for Interventional Trials) reporting guidelines (Multimedia Appendix 1) [20].

### Study Setting

**Figure 1:**
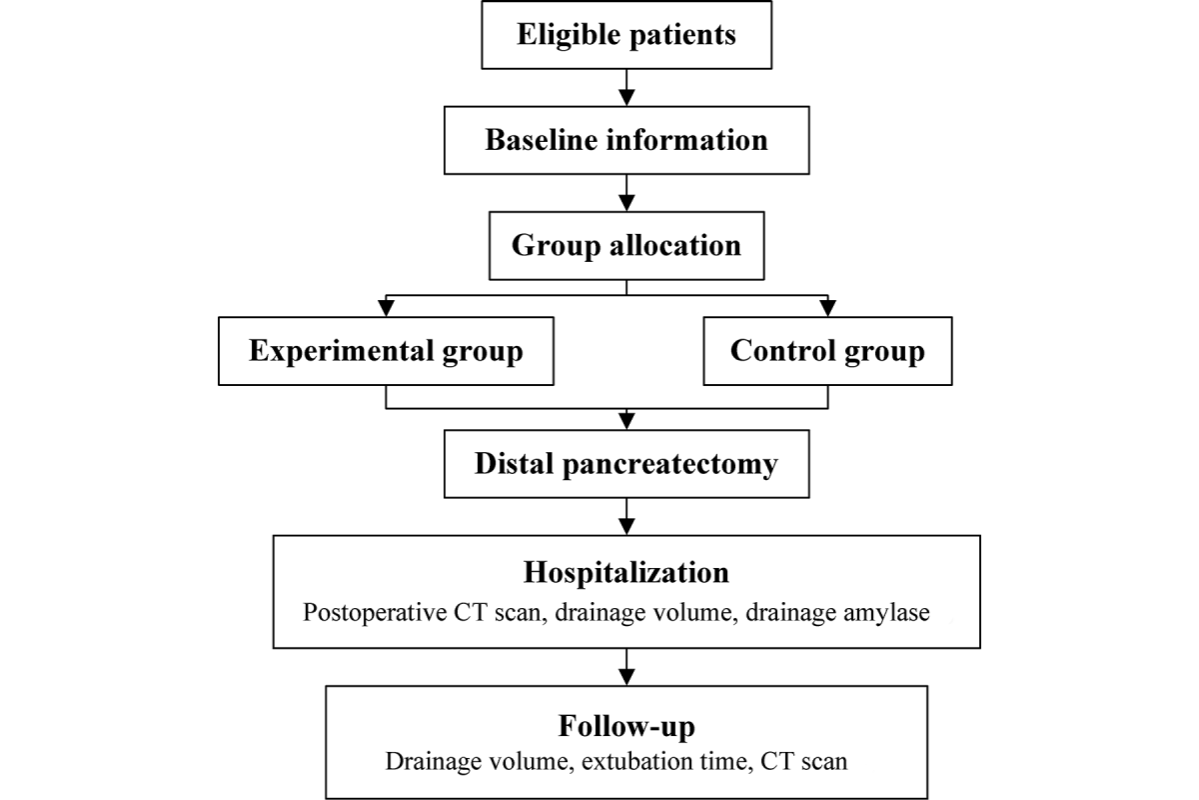
Flowchart of the study.

This nonrandomized controlled clinical study is being conducted at a tertiary center in China. The study population consists of patients who are scheduled to undergo distal pancreatectomy. Experienced surgeons will determine the eligibility of the patients and obtain informed consent from the patients (see online supplemental materials). An overview of the protocol is shown in Figure 1.

### End Points

#### Primary End Points

The primary end points are POPF and postoperative hospital stay.

#### Secondary End Points

The secondary end points are intra-abdominal infection, incision infection, and postoperative treatment costs.

### Definitions

In this study, POPF will be identified using the definition proposed by the International Study Group of Pancreatic Surgery [21]. Intra-abdominal infection and incision infection will be evaluated on the basis of the Common Terminology Criteria for Adverse Events (CTCAE) version 5.0. Severe adverse events are events rated grade 3 or higher using CTCAE. Postoperative hospital stay refers to the interval from the day of surgery to discharge. Postoperative treatment costs encompass all hospitalization expenses incurred from the patient’s return to the ward after surgery until discharge, measured in yuan renminbi.

### Patient Eligibility

#### Inclusion Criteria

The inclusion criteria were as follows.

1. Patients aged between 18 and 75 years.
2. Patients scheduled to undergo distal pancreatectomy (including the body and tail of the pancreas) with the intended pancreatic transection line located on the left side of the portal vein.
3. Patients who fully understand this study, agree to participate in it voluntarily, and sign the informed consent form.

#### Exclusion Criteria

The exclusion criteria were as follows.

1. Patients with a history of previous pancreatic surgery.
2. Patients requiring additional surgical procedures for the residual pancreas.
3. Patients with proximal pancreatic duct obstruction and planned anastomosis between the residual pancreas and the digestive tract.
4. Patients using long-acting somatostatin analogues during the perioperative period.
5. Patients who are judged by the investigator to be unsuitable for participation in this study.

### Group Allocation

Patients will be allocated to the experimental or control group according to their choice. Recruitment for either group will be discontinued upon reaching the predefined sample size.

### Study Interventions

#### Surgical Approach

The laparoscopic approach will be prioritized for all enrolled patients. Open conversion will be initiated upon the surgeon’s judgment of laparoscopic feasibility.

#### Evaluation of the Texture of the Pancreas

After the baseline visit and admission, eligible participants will be classified by the surgeon and the first assistant in terms of the texture of the pancreas (soft, medium, hard) during the operation. In case of disagreement between the surgeon and first assistant, a third surgeon will be invited to make the decision.

#### Ligation of the Pancreatic Stump for the Experimental Group

**Figure 2:**
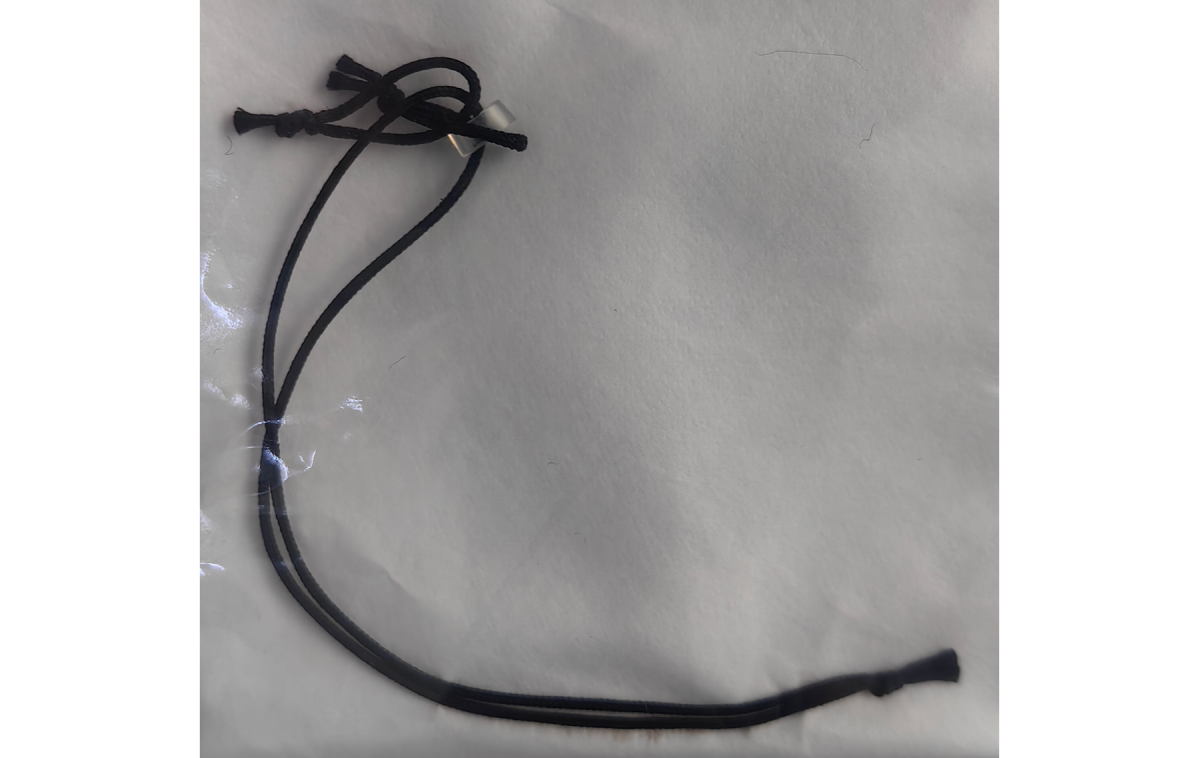
The silicone ring and the nonabsorbable silk suture used for the ligation of the pancreatic stump.

The pancreas will be ligated 5 mm from the pancreatic stump before or after severing the pancreas. To achieve proper ligation force, a predetermined force will be generated with a force device outside the body, and the deformation length of the implantable silicone/rubber will be recorded. When performing ligation in vivo, the specific ligation force will be reproduced by reproducing the deformation length measured in vitro. In this study, the ligation force will be provided by a 3.2-mm-diameter silicone ring composed of silicone and barium sulfate (Gnovo Inc). As shown in Figure 2, this silicone ring will be stretched to 15 mm, generating an applied force of 1.3 N, and the tension will be transmitted to 2 strands of size-0 braided nonabsorbable silk suture (Ethicon, Inc) during pancreatic stump ligation. No additional ligation of MPD will be performed.

#### Pancreatic Stump Management for the Control Group

The stapler technique will be the preferred method for pancreatic transection. No additional ligation of MPD will be performed. When surgeons determine that stapler use is unsuitable for a particular patient, the pancreatic stump will be managed by manual suturing. Selective ligation of the MPD will be contingent upon its identification following pancreatic transection.

#### Distal Pancreatectomy

Distal pancreatectomy will be performed according to the standard process.

#### Postoperative Management

General clinical treatment will be conducted in both groups. The patient’s body temperature and drainage volume will be recorded daily. The amount of amylase concentration in the drainage fluid as well as the blood will be routinely measured. Drains will not be removed before postoperative day 3. After postoperative day 3, drains will be left in place until the amylase concentration is less than 3 times the upper limit of normal serum amylase concentration, the output is less than 20 mL/day with an amylase concentration less than 3000 U/L, or the output is less than 5 mL/day.

#### Follow-Up

For 3 months after surgery, patients will be required to undergo regular follow-up assessments every 2 weeks through outpatient or telephone consultations. The following information will be collected during each follow-up assessment: basic information and the general condition of the patient, whether the patient has been extubated (if the patient has not been extubated, the recent drainage condition will be recorded), and recent medical interventions. Laboratory tests and imaging examinations will be performed at the surgeon’s discretion.

### Ethical Considerations

This study was approved by the ethics committee of Union Hospital, Tongji Medical College, Huazhong University of Science and Technology (approval 2024-0833-02). Written informed consent will be obtained from all patients prior to the initiation of study procedures. Participants aged 18 to 75 years will receive a comprehensive explanation of the study’s background, objectives, methodology, potential benefits, risks, and any possible inconveniences. Their rights as research participants will also be clearly outlined. All questions will be addressed thoroughly by the research team to ensure full understanding before consent is given. Participation in this study is entirely voluntary. Consent forms must be signed by the participant, or in cases involving minors or individuals unable to provide consent, by a parent or legally authorized representative. A model informed consent form has been provided and uploaded in Multimedia Appendix 2. This study will be conducted in accordance with the approved protocol and the ethical principles set forth in the current version of the Declaration of Helsinki. All participants were provided with surgical accident insurance. The research team will cover all the treatment costs for study-related complications as assessed and approved by the ethics committee. The data collected in this study will be de-identified. The results will be submitted for publication in an international peer-reviewed journal with anonymized data.

### Statistical Methods

#### Hypothesis

##### Null Hypothesis

The rate of POPF in patients who undergo pancreatic stump ligation is not lesser than that using other closure techniques.

##### Alternative Hypothesis

The rate of POPF in patients who undergo pancreatic stump ligation is lesser than that using other closure techniques.

#### Sample Size Calculation

Based on published clinical studies on the incidence of pancreatic fistula [6-8] using other closure techniques, the incidence of POPF was set at 40%. On the basis of the results of previous animal experiments, the expected incidence of POPF in the ligation strategy is 5%. Using the formula *n = 2p̅q̅(Z_α_+Z_β_)^2^/(p1-p2)^2^* for sample size calculation with the probability of type I error of .05 and the statistical power of .90, we estimated that 30 participants should be included in each group.

#### Statistical Analysis

All statistical analyses will be conducted using SAS software (version 9.3; SAS Institute). Normality tests will be conducted for measurement data, describing the mean, standard deviation, median, quartiles, minimum, and maximum values based on the distribution type. Count data will describe the number and percentage of cases in each category. The rate effect indicator will reflect the effective rate and 95% CI. Missing values will not be filled in for research purposes and will not be included in the calculation of percentages unless otherwise specified. Stratified analyses will be conducted on the basis of pancreatic texture. A logistic regression analysis for POPF will be performed to identify its independent risk factors.

## Results

The version number and date of the protocol are version 2.0 and January 22, 2025, respectively. Participant recruitment for this study commenced in February 2025 and will conclude when the estimated sample size is achieved (in or around September 2025); thereafter, the follow-up assessments will be completed (in or around December 2025). No specific funding was received for this study.

## Discussion

### Overview

POPF after distal pancreatectomy remains an unresolved issue [13,22]. The existing stump-closure techniques primarily include manual suturing and stapling, both of which carry a high risk of POPF. Animal studies suggest that the balance between pancreatic duct burst pressure and pancreatic tissue necrosis may be a key factor influencing the occurrence of POPF after distal pancreatectomy. We attempted to address this issue by ligating the pancreatic stump with an appropriate quantified amount of force.

Although suturing MPD has been shown to reduce the risk of POPF [23,24], MPD is difficult to ligate due to its location within the pancreatic parenchyma, and MPD is often difficult to locate during surgery. Therefore, the method adopted in this study involves ligating the pancreatic stump rather than MPD. Through experiments on specimens from patients with pancreatic space-occupying lesions, we found that for pancreas with soft, medium, and hard textures, ligation forces of 2.5 N using 4 strands of size-0 braided nonabsorbable silk suture or 1.3 N using 2 strands of size-0 braided nonabsorbable silk suture were sufficient to achieve a pancreatic duct burst pressure of 40-70 mm Hg. Therefore, we initiated a prospective clinical study on this pancreatic stump treatment technique, which yielded the optimal pancreatic duct burst pressure by quantifying the ligation force. The aim of this study was to determine whether this technique can reduce the risk of POPF and provide new insights into pancreatic stump closure.

Theoretically, potential surgery-related adverse events for participants of the new approach may primarily include pancreatic fistula, hemorrhage, and pancreatitis. All adverse events occurring during both the intervention and follow-up periods will be documented and submitted to the ethics committee regularly. Should the predefined criteria be met, immediate actions will include either termination of an individual participant’s trial eligibility or discontinuation of the entire study.

Pancreatic texture and patient age are important factors influencing the occurrence of POPF. To align our study with real-world clinical scenarios, we will not restrict enrollment to patients with specific pancreatic texture characteristics. Instead, we will employ a stratified analysis based on pancreatic texture during data interpretation to mitigate the potential bias arising from variations in tissue consistency. Furthermore, to reduce the confounding effect of age on the risk of POPF, we will limit participant enrollment to individuals younger than 75 years [25,26].

### Strengths of This Study

The strengths of the study can be summarized as follows. This study uses a prospective design to assess a new strategy for managing the pancreatic stump during distal pancreatectomy. The new strategy will provide a sufficiently high pancreatic duct burst pressure while preserving partial blood supply to the pancreatic stump.

### Limitations

One of the primary limitations of this study is its nonrandomized controlled design. Since this study investigates a novel pancreatic stump management technique that has not yet undergone large-scale clinical validation, the institutional ethics committee recommended an enrollment approach based on patient choice to ensure patient autonomy. Furthermore, the involvement of 2 distinct surgical techniques precluded blinding of the investigators. These factors may introduce performance and detection biases into the study. On the basis of the findings of this investigation, the research team will evaluate the need for subsequent randomized controlled clinical trials to address these methodological constraints. The relatively small sample size is another limitation of this study. The inherent variability among the included cases may prevent confirmation of the study hypothesis. We will make a data-driven decision regarding potential sample size expansion.

## Appendix

Multimedia Appendix 1 SPIRIT (Standard Protocol Items: Recommendations for Interventional Trials) checklist.

Multimedia Appendix 2 Informed consent form.

## Abbreviations

CTCAE: Common Terminology Criteria for Adverse Events
MPD: main pancreatic duct
POPF: postoperative pancreatic fistula
SPIRIT: Standard Protocol Items: Recommendations for Interventional Trials

## Data Availability

All data acquired during this trial will be made available to the involved investigators and ethics committee members for monitoring, audits, and inspections throughout the study. The study results will be published in an open-access format. Data will also be available upon reasonable request to the corresponding author via email at shanmiaogou@hust.edu.cn.
