# Supplementary material for: Ligation of the Pancreatic Stump With Quantified Force During Distal Pancreatectomy for Postoperative Pancreatic Fistula: Protocol for a Single-Center Nonrandomized Controlled Clinical Study
Source: JMIR Res Protoc. 2025 Jul 8;14:e74018. doi: 10.2196/74018 (PMC12284453; doi:10.2196/74018)
Supplement: Multimedia Appendix 2 [file resprot_v14i1e74018_app2.doc]

**Informed Consent Form**

**(Translated form Chinese)**

| Project Name | Ligation of pancreatic stump with quantified force during distal pancreatectomy for postoperative pancreatic fistula |
| --- | --- |
| Protocol Number | PS-PF |
| Protocol Number | 2.0 |
| Version Date | 2025.1.22 |
| Principal Investigator | Dr. Shanmiao Gou |
| Department | Pancreatic Surgery |

Dear ：

You are being invited to participate in a clinical study initiated by Prof. Gou Shanmiao, titled "Ligation of pancreatic stump with quantified force during distal pancreatectomy for postoperative pancreatic fistula." The following sections describe the background, purpose, methods, potential benefits, risks, and inconveniences associated with this study, as well as your rights as a participant. Please read this document carefully before deciding whether to participate. This informed consent form provides you with the necessary information to make an informed decision. If you have any questions, please ask the research team to ensure you fully understand the content. Participation in this study is entirely voluntary. If you agree to participate, please sign the declaration section of this informed consent form.

This study has been approved by the Ethics Committee of Union Hospital, Tongji Medical College, Huazhong University of Science and Technology.

**Background**

Distal pancreatectomy (DP) is a standard surgical procedure for treating inflammatory lesions and benign or malignant tumors in the body and tail of the pancreas. Postoperative pancreatic fistula (POPF) is a common and serious complication of DP, with an incidence rate of approximately 30–50%. POPF can lead to delayed gastric emptying (DGE), intestinal obstruction, bleeding, wound infection, intra-abdominal abscess, and sepsis. These complications may prolong hospital stays, increase medical costs, and potentially result in death. POPF is associated with various risk factors, among which pancreatic thickness and fat content are key determinants. Additionally, factors such as age, height, body mass index (BMI), pancreatic texture, operative time, blood loss, smoking, trauma, and the preoperative C-reactive protein-to-albumin ratio can influence the incidence and severity of POPF. Currently, there is no established strategy to reduce the risk of pancreatic fistula.

Besides manual suturing or stapler closure of the pancreatic stump, ligation has been performed proximal to the pancreatic stump to provide partial pancreatic duct burst pressure, thereby reducing the risk of pancreatic fistula. However, controlling the ligation force is challenging: excessive force may cause tissue necrosis and pancreatic duct regeneration, while insufficient force may fail to provide adequate burst pressure. This study aims to apply quantified ligation force to provide sufficient burst pressure while avoiding tissue necrosis, thereby reducing the risk of pancreatic fistula.

**Aim**

To determine whether applying quantified ligation force to pancreatic stump can reduce the risk of pancreatic fistula.

**Eligibility Criteria**

*Inclusion criteria:*

1. Patients fully understand this study, voluntarily participate, and sign the Informed Consent Form (ICF);

2. Age between 18 and 75 years old;

3. Planned for distal pancreatectomy (including body and tail of the pancreas);

4. The intended pancreatic transection line is located on the left side of the portal vein.

*Exclusion criteria:*

1.History of previous pancreatic surgery;

2.Additional surgical procedures required for the residual pancreas;

3.Proximal pancreatic duct obstruction, with planned anastomosis between the residual pancreas and the digestive tract;

4.Use of long-acting somatostatin analogues during the perioperative period;

5.Patients who are judged by the investigator to be unsuitable for participation in this study.

**Study Overview**

This is a single center non-randomized controlled clinical study. Approximately 30 patients scheduled for distal pancreatectomy will be enrolled in each group. After distal pancreatic resection, the pancreas was ligated at 5 mm from the pancreatic stump with a quantified force. The study consists of a screening period (preoperative phase) and a follow-up period. Participants will sign the informed consent form and undergo baseline assessments during the screening period. Eligible patients will be enrolled, and all participants will complete the required examinations during treatment to evaluate safety and efficacy.

**Biological Sample Collection**

*Complete Blood Count (CBC)*

Includes white blood cells, neutrophils, eosinophils, basophils, monocytes, lymphocytes, red blood cells, hemoglobin, cytokines, and lymphocyte subsets. Performed during the screening period and at the end of treatment. Frequency during postoperative treatment will be determined based on the patient's condition.

*Blood Biochemistry*

Includes fasting blood glucose, glycated hemoglobin, procalcitonin, C-reactive protein, alanine aminotransferase (ALT), aspartate aminotransferase (AST), alkaline phosphatase (ALP), total bilirubin, direct bilirubin, total protein, albumin, lactate dehydrogenase (LDH), blood urea nitrogen (BUN), serum creatinine, potassium, sodium, chloride, and calcium. Frequency during postoperative treatment will be determined based on the patient's condition.

*Body Fluid Biochemistry*

Drainage fluid amylase. Frequency during postoperative treatment will be determined based on the patient's condition.

*Urinalysis*

Includes urine white blood cells, red blood cells, and protein. Frequency during postoperative treatment will be determined based on the patient's condition.

*Coagulation Function Tests*

Frequency during postoperative treatment will be determined based on the patient's condition.

**Biological Sample Storage and Disposal**

Not applicable. This study does not alter the standard clinical management of distal pancreatectomy and does not generate additional biological samples.

**Potential Risks of Participation**

Ligation of the pancreatic stump are common methods for managing the pancreatic stump. Theoretically, the risk of postoperative pancreatic fistula in this study will not be higher than that of ligation with non-quantified force, and the risk of pancreatic stump necrosis will not be higher than that of ligation with non-quantified force.

Ligation of the pancreatic stump with quantified force does not rule out the possibility of a higher risk of postoperative pancreatic fistula or pancreatic stump necrosis and infection.

Any treatment may be ineffective or result in complications due to the disease. If your doctor identifies such situations during the trial, they may recommend alternative treatments. Your doctor will discuss specific treatment options with you based on your condition.

**Potential Benefits of Participation**

Participation in this study may or may not provide therapeutic benefits. You will receive closer follow-up and monitoring from your doctor. The information obtained from this study will contribute to a better understanding and treatment of this condition.

You will be responsible for the costs of routine tests (e.g., CBC, blood biochemistry, blood glucose) included in this study. No additional specialized medical tests are involved.

**Compensation for Study-Related Injuries**

In the event of a serious adverse event related to the study, compensation will be provided in accordance with insurance terms and legal requirements, provided that the clinical trial protocol is followed.

**Costs to Participants**

You will be responsible for the costs of routine tests (e.g., CBC, blood biochemistry) included in this study. No additional specialized medical tests are involved.

**Handling of New Clinical Research Information**

If new information arises that may affect your decision to continue participating in the study, the investigator will promptly inform you and your family.

**Circumstances for Study Termination**

1. You may terminate your participation after completing all study procedures under the guidance of the research team.
2. You may withdraw from the study at any time if:

a. You withdraw your consent.

b. Your disease progresses or you pass away.

c. You are lost to follow-up.

d. The investigator determines that termination is in your best interest.

e. You fail to comply with study requirements, and the investigator deems it necessary to terminate your participation.

f. The sponsor or regulatory authorities require the study to be terminated.

1. You may voluntarily withdraw at any time.

**Duration of Participation**

The study includes a screening period and a follow-up period. Participants will sign the informed consent form and undergo baseline assessments during the screening period. Eligible patients will be enrolled and complete the required examinations during treatment to evaluate safety and efficacy. The follow-up period is 6 months post-surgery, or until the disease and complications are completely resolved.

**Number of Participants**

This is a small-sample exploratory study. Based on sample size calculations, 30 participants will be enrolled in each group in this phase.

**Privacy and Confidentiality**

Your research data will be stored at Union Hospital, Tongji Medical College, Huazhong University of Science and Technology. The investigator, research authorities, and ethics review board may access your medical records. Any public reports of the study results will not disclose your identity. We will make every effort to protect the privacy of your medical information within the limits of the law.

**Voluntary Participation and Right to Withdraw**

Participation in this study is entirely voluntary. You may refuse to participate or withdraw from the study at any time without discrimination or retaliation, and your medical care and rights will not be affected. If you decide to withdraw, please contact your doctor to ensure proper medical care.

**Assistance During the Study**

If you have any questions about the study, its progress, or your rights as a participant, or if you experience any discomfort or harm related to the study, you may contact the investigator, Prof. Gou Shanmiao, at 13419693160, or the Ethics Committee of Union Hospital, Tongji Medical College, Huazhong University of Science and Technology, at 027-85726375.

**Participant Declaration**

I have carefully read this informed consent form and have had the opportunity to ask questions, all of which have been answered to my satisfaction. I understand that participation in this study is voluntary, and I may choose not to participate or withdraw at any time without discrimination or retaliation. My medical care and rights will not be affected.

If I require other diagnostic/treatment options, fail to comply with the study plan, or have other valid reasons, the investigator may terminate my participation in this clinical study.

I voluntarily agree to participate in this clinical study and will receive a signed copy of this informed consent form.

I voluntarily choose to participate in the (experimental group/control group) of this study.

Participant Name (Printed):

Participant Signature:

Date:

If the participant is unable to sign due to incapacity or is a minor, a guardian must sign.

Guardian Name (Printed):

Guardian Signature:

Date:

Relationship to Participant:

Reason for Participant's Inability to Sign:

If the participant or guardian is unable to read, an impartial witness must sign.

Impartial Witness Name (Printed):

Impartial Witness Signature:

Date:

**Investigator Declaration**

I have accurately explained the contents of this informed consent form to the participant and answered all their questions. The participant voluntarily agrees to participate in this clinical study.

Investigator Name (Printed):

Investigator Signature:

Date:
